# Traditional and Non-Conventional Pasta-Making Processes: Effect on In Vitro Starch Digestibility

**DOI:** 10.3390/foods10050921

**Published:** 2021-04-22

**Authors:** Rossella Dodi, Letizia Bresciani, Beatrice Biasini, Marta Cossu, Francesca Scazzina, Federica Taddei, Maria Grazia D’Egidio, Margherita Dall’Asta, Daniela Martini

**Affiliations:** 1 Department of Food and Drug, Human Nutrition Unit, University of Parma, 43125 Parma, Italy; rossella.dodi@unipr.it (R.D.); letizia.bresciani@unipr.it (L.B.); beatrice.biasini@unipr.it (B.B.); cossu.marta@gmail.com (M.C.); francesca.scazzina@unipr.it (F.S.); 2 CREA Research Centre for Engineering and Agro-Food Processing, 00189 Rome, Italy; federica.taddei@crea.gov.it (F.T.); mariagrazia.degidio@gmail.com (M.G.D.); 3 Department of Animal Science, Food and Nutrition, Università Cattolica del Sacro Cuore, 29122 Piacenza, Italy; 4 Department of Food, Environmental and Nutritional Sciences (DeFENS), Università Degli Studi di Milano, 20133 Milan, Italy; daniela.martini@unimi.it

**Keywords:** slowly digestible starch, carbohydrates, fiber, in vitro digestion, micronization

## Abstract

Pasta is a carbohydrate-rich food with a low glycemic index (GI) and is one of the main sources of slowly digestible starch (SDS). The presence of bran fractions (BFs) in pasta may enhance its health potential, owing to the content of fiber, micronutrients, and bioactive compounds; however, at the same time, BF may affect starch digestibility. In this study, the bioaccessibility of starch in pasta made with BF-enriched semolina (BF pasta), or only with micronized debranned kernel (DK pasta), and a control pasta made with traditional semolina was evaluated by applying two different in vitro models. The control pasta showed a percentage of SDS about four-fold higher than that of the BF pasta and 1.5-fold higher than that of the DK pasta (*p* < 0.05). The amount of starch released during simulated gastrointestinal digestion was slightly lower, but not significantly different, for the control pasta than for both the BF and DK pasta. These results suggest that the presence of a higher amount of dietary fiber in BF pasta can affect the structure of the food matrix, interfering with the formation of the gluten network, water absorption, and starch granule accessibility, while micronization could enhance starch digestibility due to starch gelatinization. These findings emphasize the need to optimize the process for producing fiber-rich pasta without affecting its low starch digestibility and, consequently, its GI.

## 1. Introduction

Pasta is one of the staple foods of the Mediterranean diet, and it is widely produced and consumed all over the world [1]. In 2019, in Italy—where pasta represents one of the key foods of the gastronomic tradition—consumption reached about 23 kg/per capita per year [1]. Depending on the total energy intake level, Italian dietary guidelines suggest the daily consumption of 3.5 to 6 portions of carbohydrate-rich foods, such as pasta, rice, and other cereals or cereal-derived products [2], which contribute to the intake of complex carbohydrates both in Italy and in many other countries, even those outside the Mediterranean basin [1,3]. Due to its wide consumption, pasta contributes to covering the recommended 45–60% of the total daily energy intake from carbohydrates and, if consumed as wholegrain pasta, the suggested dietary target for fiber (at least 25 g per day) [4]. 

Besides available starch, pasta—especially when consumed as whole-wheat pasta—is indeed a good carrier of fiber, micronutrients, and bioactive compounds [5,6]. In particular, whole-wheat semolina is rich in fiber, mainly insoluble fiber (e.g., cellulose, arabinoxylans), which is well-recognized for its beneficial role in bowel health and many other health outcomes, including a positive role in metabolic health and, thus, in the prevention of several chronic diseases [6,7,8]. Despite these beneficial effects associated with wholegrain products, the consumption of whole-wheat pasta in Italy still seems limited [9], even though recent data showed a substantial increase in the past decade of launches on the market and the consumption of wholegrain products [10]. Potential barriers to the consumption of wholegrain foods include personal, product-specific, and external factors, such as sensory aspects and dietary habits [11,12]. In particular, inadequate knowledge of the positive effect of wholegrain consumption on chronic disease risk reduction often leads consumers to prefer refined grain products [13,14]. 

The food structure of pasta is the result of changes occurring in its main components, namely, starch and protein, throughout the technological production process [15], which, in turn, influences the nutritional quality of this product. Indeed, pasta processing leads to an increase in the slowly digestible starch (SDS) fraction in the final product, which elicits a lower post-prandial glycemic response after consumption compared to that from other cereal-based products, such as rice and bread [16]. To date, this is one of the main evidenced reasons behind the beneficial effect of pasta consumption [17,18,19]. The use of innovative technological processes has been proposed as a strategy to produce cereal-based products such as pasta that preserve the natural health properties of grains while limiting the negative aspects related to the use of wholegrain products [20]. Among these strategies, the use of debranning has been proposed. Debranning consists of pearling and pealing of the kernels that can be used to obtain both selected bran fractions (BFs) and debranned kernels (DKs) [21,22]. Different studies have demonstrated that debranning is a pretreatment potentially able to improve milling yields [23]; moreover, the use of debranning products allows for the making of pasta with a high content of dietary fibers, vitamins, and phenolic compounds and minimal effects on sensory properties, using only the natural endowment of durum wheat [21,24,25]. Additionally, preprocessing could reduce the total microbial contamination and the content of mycotoxins or heavy metals in flour, which, in turn, affect its safety and quality [23,26,27,28]. 

However, the modification of process parameters may also affect the pasta structure and potentially change the digestibility of the starch and protein fractions [29,30]. For instance, the addition of BF or the use of a different process in pasta-making can affect starch digestibility. In fact, the presence of bran within the wholemeal pasta matrix may physically interfere with the gluten matrix, making the structure highly porous, which, in turn, increases the accessibility of the starch granules to α-amylase during digestion [15]. Therefore, investigating the starch digestibility in vitro is of physiologic relevance and represents a useful approach for predicting the in vivo bioavailability of carbohydrates contained in pasta and the glycemic index (GI) [31,32].

Thus, because the modification of process parameters may influence the digestibility of starch, influencing both the accessibility to the digestive enzymes and, consequently, the glycemic response in vivo, the aim of the present study was to investigate the in vitro digestibility of starch in pasta produced by using BFs or DKs and to compare it with that of a traditional pasta made with semolina.

## 2. Materials and Methods

### 2.1. Chemicals

Reagents and enzymes were purchased from Sigma-Aldrich (St. Louis, MO, USA), unless otherwise noted. The enzymes used are reported as follows: pepsin (EC number 3.4.23.1), pancreatin (EC number 232-468-9), guar (EC number 232-536-8), invertase (EC number 3.2.1.26), amyloglucosidase (AMG) from E-AMGDF Megazyme kit (Wicklow, Ireland).

### 2.2. Debranning and Traditional Milling Processes

The Italian *Triticum durum* wheat Normanno, a widely used Italian durum wheat cultivar, was used as raw materials for the whole experiment. In detail, an aliquot of kernels was debranned three sequential times for about 90 s each, using a pilot plant (NAMAD, Rome, Italy), to obtain three bran fractions (BFs 1, BFs 2, BFs 3) and aliquots of the resulting kernels (DKs 1, DKs 2, DKs 3), corresponding to ranges of debranning levels (DLs) of 0–2.80%, 2.81–5.10%, and 5.11–8.00%, respectively. An additional aliquot of the non-debranned kernels of the same cultivar was traditionally milled in a pilot plant (Buhler MLU 202, Uzwil, Switzerland) to obtain semolina. 

### 2.3. Pasta Samples: Preparation and Cooking

Some of the debranning products described in Section 2.1 (i.e., BF2 and DK1) and semolina were used to produce two different pasta samples: (i) BF pasta, produced by enriching semolina with BFs 2 (BFs 2: semolina ratio of 30:100 *w*/*w*); and (ii) DK pasta, produced by using only micronized DKs 1 that still include BF2 used for the BF pasta. DK 1 was micronized using a KMX-500 micronizer (Separmicrosystem S.a.S, Brescia, Italy). The third pasta type (control pasta, CTRL), used as a reference, was made by using only semolina obtained as described in Section 2.2. The three samples were processed into spaghetti by an experimental press (NAMAD, Rome, Italy) and were dried using an experimental drier (AFREM, Lyon, France) for 20 h, applying a low-temperature drying process (50 °C). The pasta-making process was repeated twice. A cooking test was performed by adding 100 g of dried pasta to 1 L of boiling tap water with a standard cooking time of 13 min. Exhaustive information on the technological process and nutritional composition of the pasta samples was reported in a previous study [24]. 

### 2.4. Available Starch Determination

Determination of the available starch (Av starch) was carried out using the AOAC Method 2002.02, AACC Method 32-40.01 (Megazyme assay kit, K-RSTAR). The available starch analysis was performed according to the manufacturer’s instruction, with slight modifications.

Briefly, 150 mg of cooked and minced pasta was weighed and incubated in a Dubnoff bath (ISCO, Milan, Italy) with pancreatic α-amylase and AMG for 16 h at 37 °C and 180 strokes/min. After the incubation, 4 mL of 100% ethanol was added to each sample and centrifuged at 3000 rpm for 10 min. After the centrifugation, the supernatant was transferred into a 100 mL matrass. The tubes containing the pellet were washed three times with 50% aqueous ethanol, dissolved in 2 mL of KOH (2 M), and put on ice. After 20 min, 8 mL of sodium acetate buffer (1.2 M, pH 3.8) was added into the samples, and after the addition of 100 μL of AMG (3300 U/mL), the samples were incubated at 50 °C for 30 min. The samples were centrifuged for 10 min at 3000 rpm and stored at −20 °C until the resistant starch analysis. 

A volume of 100 mL of sodium acetate buffer (100 mM, pH 4.5) was added into the matrass containing the supernatant to adjust the total volume. Quantities of 200 μL of the solution were transferred into tubes, and 20 μL of AMG (300 U/mL) was added. The tubes were incubated for 20 min at 50 °C, and the available starch was quantified in the supernatant of the samples by means of an automatic glucose analyzer (model 2900, Yellow Springs Instrument Company, Yellow Springs, OH, USA). The analyses were performed in triplicate for each sample.

### 2.5. In Vitro Starch Digestibility

In vitro digestion of the pasta was performed following the method of Brighenti and colleagues [32], with some modifications [33]. Samples were cooked and extruded through 7 mm holes of a hand-operated mincer (Sirius, Karl Krüger). Briefly, 8 g samples were weighed and suspended in 5 mL of preheated (37 °C) 20 mM sodium phosphate buffer (pH 6.9, 10 mM NaCl) and 25 mL of preheated (37 °C) 0.9% NaCl with 1.5 mL of human saliva. Saliva was collected from three non-smoking adult donors after careful tooth brushing and abstinence from food and drink for at least 1 h prior to the experiment. After 2 min incubation in a shaking water bath (SW23, Julabo, Milan, Italy) at 37 °C and 160 strokes/min, the pH was adjusted to 2–2.5 using 5 M HCl. One milliliter of a solution of 0.9% NaCl dissolved porcine pepsin (2500 U/mL pepsin, was then added to each sample in order to mimic the gastric phase. The mixtures were incubated in a shaking water bath at 37 °C and 200 strokes/min for 2 h. Intestinal digestion was simulated by correcting the pH of the samples with 5 M NaOH to 6.9 and adjusting the volume to 50 mL with the addition of 20 mM sodium phosphate buffer (pH 6.9, 10 mM NaCl). After the addition of 100 mg of pancreatin from a porcine pancreas, each sample was transferred into a dialysis tube (12–14 kD, Spectra/Por) with 5 glass marbles. The tubes were sealed and suspended in sealed containers with 600 mL of 20 mM sodium phosphate buffer (pH 6.9, 10 mM NaCl). 

The containers were incubated for 5 h in a shaking water bath at 37 °C and 200 strokes/min to simulate the intestinal phase. The dialysate (1 mL) was collected after 15, 30, 45, 60, 90, 120, 150, 180, 240, and 300 min from the start of incubation. Complete starch hydrolysis was carried out by adding 30 μL of 0.5 N acetic acid and 20 μL of a solution of AMG from *Aspergillus niger* (300 U/mL AMG in water) to 0.5 mL of dialysate and incubating the samples at 60 °C for 2 h. At the end of the intestinal phase, the glucose concentration derived from starch hydrolysis was quantified using an automatic glucose analyzer (model 2900, Yellow Springs Instrument Company, Yellow Springs, OH, USA). The rate of digested starch from the samples (expressed as the percentage of digested starch) was calculated for each time point as follows: % digested starch = (glucose concentration × 0.9/Av starch) × 100. In vitro digestions were performed in triplicate for each product.

### 2.6. Slowly Digestible Starch and Rapidly Digestible Starch Determination

The percentages of slowly and rapidly digestible starch (SDS and RDS, respectively) were analyzed according to the method proposed by Englyst and colleagues [31], with slight modifications. Briefly, samples were cooked and extruded through 7 mm holes of a hand-operated mincer (Sirius, Karl Krüger), and then 2 g of product underwent several enzymatic attacks. After adding 10 mL of pepsin–guar solution (5 g/L pepsin and 5 g/L guar in 0.05 M HCl), the samples were vortex-mixed and incubated in a shaking water bath (SW23, Julabo) at 37 °C and 180 strokes/min for 30 min. Ten milliliters of preheated (37 °C) 0.25 M sodium acetate were added to 5 glass marbles, and the tubes were mixed and placed in a water bath for 3 min to equilibrate the temperature. The enzyme mixture was prepared by dissolving 3.3 g of pancreatin in 22 mL of distilled water, and the tubes were centrifuged (3200 rpm for 10 min). The supernatant (15 mL) was collected; then, 3.6 mL of AMG and 37.5 mg of invertase from baker’s yeast (*Saccharomyces cerevisiae*) were diluted in 3.06 mL of distilled water and added to the supernatant. Five milliliters of the enzyme mixture were added to each sample, and the samples were incubated in a water bath at 37 °C and 200 strokes/min. After 20 min and 120 min, 1 mL of hydrolysate was centrifuged (14,000 rpm for 5 min), and the supernatant was diluted in distilled water (diluted 1:10), then used to determine the total glucose concentration (G_20_ and G_120_, respectively).

To determine the free sugar glucose (FSG), the method of Englyst and colleagues [31] was used with some modifications. Briefly, 2 g of each product was extruded through 8 mm holes of a hand-operated mincer (Sirius, Karl Krüger) and weighed into plastic flasks. After the addition of 25 mL of 0.1 M sodium acetate buffer (pH 5.2) and 5 glass marbles, samples were vortex-mixed and incubated in a water bath (SW23, Julabo) at 100 °C for 30 min. Samples were vortex-mixed again and cooled to 37 °C; then, 0.153 mL of invertase solution (12.3 mg/mL invertase from baker’s yeast (*S. cerevisiae*) in water) was added to the samples before incubation at 37 °C and 200 strokes/min for 30 min. One milliliter of hydrolysate was collected and centrifuged (14,000 rpm for 5 min), and the supernatant was used to determine the FSG.

The levels of rapidly available glucose (RAG), slowly available glucose (SAG), RDS, SDS, and Av starch were calculated as described by Englyst and colleagues [34].

The glucose amounts released after 20 and 120 min and after FSG analysis were quantified by means of an automatic glucose analyzer (model 2900, Yellow Springs Instrument Company, Yellow Springs, OH, USA). The analyses were performed in quadruplicate for each sample.

### 2.7. Statistical Analysis

All the results are expressed as the mean ± standard deviation (SD). The data distribution was assessed by means of the Shapiro–Wilk test, and the differences among results were studied by analysis of variance through one-way ANOVA and Bonferroni post hoc testing. Statistical significance was determined at *p* < 0.05, and the analyses were performed using SPSS Statistics software (version 26, IBM, Armonk, NY, USA).

## 3. Results

### 3.1. Starch Digestibility of Pasta Samples

**Figure 1:**
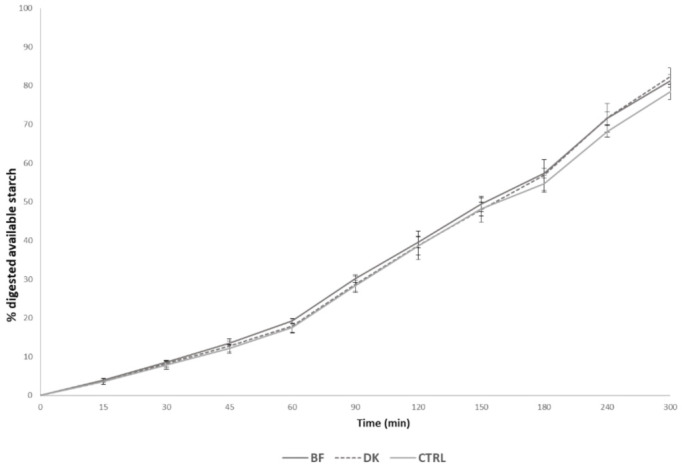
Lines represent the digested starch reported as a percentage of the total available starch during the in vitro digestion of each test food. Values are reported as the mean ± standard deviation (SD) (*n* = 3). BF: pasta produced by enriching semolina with a durum wheat bran fraction; CTRL: control, pasta produced by traditional milling; DK: pasta produced by using micronized debranned kernels. Statistical analysis was performed via one-way ANOVA and Bonferroni post hoc testing (*p* < 0.05).

The percentages of digested starch during the 5 h intestinal phase digestion are shown in Figure 1, with a focus on the percentages of starch digested after 120 and 300 min (Figure 1). After 120 min of simulated digestion, the BF pasta presented the highest percentage of starch digestion (39.67 ± 1.54%), followed by the DK (38.77 ± 3.63%), while the CTRL pasta showed the lowest percentage (38.66 ± 2.32%). After 300 min of digestion, the different starch digestibility percentages were 81.24 ± 1.62%, 82.44 ± 2.09%, and 78.42 ± 2.02% for BF, DK, and CTRL, respectively. Although a trend of reduced starch release during the digestion of CTRL compared to the two pasta samples produced via a non-conventional process was observed, no statistically significant differences among the samples were evident (*p >* 0.05).

### 3.2. Slowly and Rapidly Digestible Starch Determination

The values of RAG, SAG, RDS, SDS, Av starch, and FSG are reported in Table 1. BF had the highest amounts of RAG (17.81 ± 0.91 g/100 g) and RDS (15.71 ± 0.99 g/100 g) compared to the DK and CTRL samples. On the contrary, the CTRL pasta showed the highest values for SAG, SDS, and Av starch (8.94 ± 1.23, 8.04 ± 1.11, and 20.16 ± 0.98 g/100 g, respectively).

**Figure 2:**
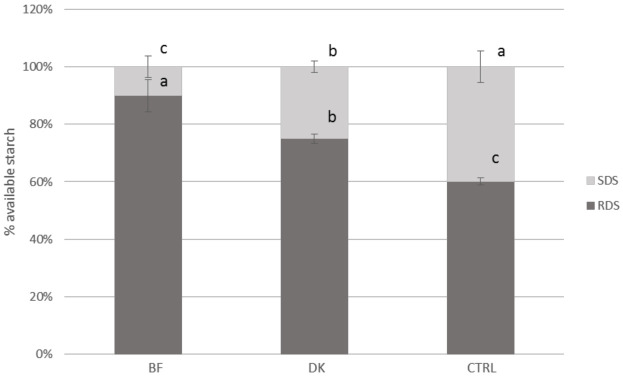
RDS and SDS expressed as percentages of the available starch (mean ± SD) (*n* = 4) for the pasta samples. BF: pasta produced by enriching semolina with a durum wheat bran fraction; CTRL: control, pasta produced by traditional milling; DK: pasta produced by using micronized debranned kernels; RDS: rapidly digestible starch; SDS: slowly digestible starch. Statistical analysis was performed via one-way ANOVA and Bonferroni post hoc testing. Different letters indicate statistical significance (*p* < 0.05).

The percentage contributions of RDS and SDS to the Av starch (considered as 100%) are graphically reported in Figure 2. The ratio between SDS and Av starch was different among the three samples; in fact, the control (CTRL) showed a percentage four-fold higher than that for the BF and 1.5-fold higher than that for the DK sample.

## 4. Discussion

The present study aimed at evaluating the in vitro digestibility of three different pasta samples made from the cultivar Normanno using different types of pasta-making processes. In particular, this study explored the impact of the use of debranned products on the starch digestibility of pasta by applying two different methods for investigating the digestibility of starch, both recognized as suitable for assessing starch digestibility in food products [35]. The results of the present work reveal that the CTRL pasta showed the highest value of SDS/Av starch and the lowest value of RDS/Av starch, which could reflect a lower glycemic response in vivo compared to the BF and DK samples [36]. The process applied for enriching the pasta with fiber and bioactives seems ineffective in maintaining a compact starch matrix. This could be ascribable to the different process applied for the production of pasta samples. This may lead to the presence of a high amount of dietary fiber in BF pasta that could affect the structure of the food matrix, interfering with the formation of the gluten network, water absorption, and starch granule accessibility [37]. The DK pasta showed intermediate values of SDS/Av starch and RDS/Av starch, which could be due to the smaller size of bran particles. Moreover, it was previously shown that micronized samples of barley and maize pasta exhibited increased starch digestibility, and this could be attributed to starch gelatinization during micronization without significant retrogradation during storage [38,39]. When the method proposed by Brighenti and colleagues was applied, the same trend of starch digestibility was observed, with the CTRL showing a slightly lower starch release during digestion, even though no statistically significant differences were observed. However, the differences obtained between the two in vitro methods could be ascribable to the difference in the oral phase and in the incubation system [40]. Starch bioaccessibility is indeed influenced by, among other things, the mastication process. Through mastication, food is broken into smaller pieces, and the rate of digestion also depends on the time and the intensity of chewing, mainly due to the contact of the food surface with saliva α-amylase, responsible for the first starch hydrolysis [41]. However, the structural properties of pasta products can lead to different breakdown patterns during mastication and, consequently, different in vitro digestibility [42]. 

The debranning process improves the yield and degree of semolina refinement and enhances the nutritional value of the end-products [21], allowing us to obtain BFs with high fiber and bioactive contents. This process was used in combination with micronization, a technological process which enables a reduction in the food matrix into a fine powder, improving the bioaccessibility of the bioactive compounds [43] and making the bran particles smaller, lowering the impact of the dietary fiber on the gluten matrix. However, several studies have demonstrated the impact of fiber on the rate of starch digestion, with non-starch polysaccharides being responsible for discontinuity in the network, leading to faster hydrolysis. The addition of fiber to durum wheat pasta can interfere with the gluten structure, thus disrupting the continuity of the protein−starch matrix and making the starch granules more susceptible to enzymatic degradation [37]. In addition to fiber, starch digestibility in cereal-based products can be affected by several other factors, including the type and source of starch, the presence of protein matrixes, and the processing method [44].

In a previous study [24], pasta produced with debranning products (DK and BF) presented higher contents of phenolic compounds and other bioactives compared to traditional pasta, with minimal effects on its sensory properties [25]. However, the effect on starch digestibility related to the presence of debranning products has not been investigated. Bioactives present in foods may also play a key role in reducing the post-prandial glycemic response of carbohydrate-rich foods in vivo [45]. This can be mediated by the direct inhibition of starch enzymatic digestion, but also by other physiological mechanisms, such as inhibition of the absorption and potentially increased insulin release at the β-pancreatic level [45]. Therefore, we can hypothesize that a higher amount of bioactives in BF and DK samples may lead to a reduced glycemic response in vivo, which is strictly dependent on the bioaccessibility of phenolic compounds. 

Diets exerting a low glycemic response favorably affect glucose metabolism and health status [46] and have been associated with a lower risk of many chronic diseases, such as type 2 diabetes and other cardiometabolic diseases, compared to high-GI diets [18,46,47]. In this scenario, pasta is a milestone in several healthy eating patterns, such as the Mediterranean diet, and its consumption is associated with several health benefits [17,18,19,48]. The consumption of pasta has decreased in recent years, probably because its consumption is wrongly associated with the myth of weight gain from the consumption of carbohydrate-rich foods [49]. However, recent studies have investigated the effect of pasta consumption on body weight and disease risk. Several publications have emphasized the beneficial role of pasta consumption on the obesity epidemic and cardiometabolic risk factors both in healthy subjects and in obese and diabetic patients [20,21,22,37]. In particular, pasta consumption, in the context of a low-GI diet, has shown to be involved in the reduction in body weight and markers of adiposity, such as waist circumference and waist-to-hip ratio [19]. Similar results emerged from another clinical trial recently conducted on obese patients [17]. In this study, the consumption of a hypocaloric diet characterized by an intake of at least five portions per week of pasta led to a higher reduction in body weight compared to the consumption of a lower amount of pasta (≤3 portions/week). Finally, patients with type 2 diabetes did not show worse glucose control, measures of adiposity, or major cardiovascular risk factors when pasta was included in their diet within the recommended consumption amount [48]. Based on this evidence, the present work focused on the importance of producing high-quality pasta, taking into account several nutritional factors.

Finally, it is worth noting that “pasta” as a category includes a large number of heterogeneous types of products which differ in shape, ingredients, and nutritional composition, eliciting different responses in humans [50]. Considering the crucial role of pasta in several dietary patterns and the overall high worldwide consumption, it is worth investigating strategies for maximizing the nutritional quality of this product, preserving its naturally high SDS content. Therefore, the investigation of starch digestion in non-conventional pasta-making processes seems to be a potential strategy to obtain pasta with high contents of SDS, fiber, micronutrients, and bioactives to increase its health-related beneficial properties. 

## 5. Conclusions

The starch digestibility of the BF and DK pasta samples after in vitro simulated gastrointestinal digestion was affected by the non-conventional pasta-making process applied to the samples, as compared to the traditional process. Enriching pasta with bran fractions seems to affect starch digestibility by decreasing the SDS content, while this effect was less obvious in pasta made with micronized debranned kernels. However, the traditional pasta-making process led to the product with the highest amount of SDS. This result may depend on the different structural properties of the three pasta samples, leading to differences in the rate of starch digestion in vitro. Therefore, further investigations should focus on the evaluation of the microstructure of pasta samples, and in vivo studies are needed to clarify the role of debranning products and the micronization process on the GI of pasta products. Moreover, future studies should also investigate the bioaccessibility and bioavailability of polyphenolic compounds from BF and DK pasta samples, to obtain a clearer picture of the overall nutritional quality of these products. Considering that dietary guidelines suggest the consumption of low-GI and high-fiber foods, it is strongly advisable to further explore new technologies for preserving the low impact of pasta on post-prandial glycaemia but guaranteeing the presence of fiber, the global consumption of which is still lower than the recommendations.

## Figures and Tables

**Table 1:** The RAG, SAG, SDS, RDS, Av starch, and FSG values of the pasta samples assessed by an in vitro method. Values are expressed as the mean ± SD (*n* = 4).

Pasta Products	RAG (g/100 g)	SAG (g/100 g)	SDS (g/100 g)	RDS (g/100 g)	Av Starch (g/100 g)	FSG (g/100 g)
BF	17.81 ± 0.91 ^a^	1.95 ± 0.72 ^c^	1.75 ± 0.65 ^b^	15.71 ± 0.99 ^a^	17.46 ± 0.37 ^b^	0.36 ± 0.01 ^b^
DK	14.88 ± 0.32 ^b^	4.78 ± 0.39 ^b^	4.30 ± 0.35 ^b^	12.91 ± 0.28 ^b^	17.21 ± 0.21 ^b^	0.54 ± 0.01 ^a^
CTRL	13.77 ± 0.27 ^b^	8.94 ± 1.23 ^a^	8.04 ± 1.11 ^a^	12.11 ± 0.24 ^b^	20.16 ± 0.98 ^a^	0.31 ± 0.00 ^b^

Av starch: available starch; BF: pasta produced by enriching semolina with a durum wheat bran fraction; CTRL: control, pasta produced by traditional milling; DK: pasta produced by using micronized debranned kernels; FSG: free sugar glucose; RAG: rapidly available glucose; RDS: rapidly digestible starch; SAG: slowly available glucose; SDS: slowly digestible starch. Data in the same column with different letters indicate significant differences at *p* < 0.05, according to Bonferroni post hoc testing.
